# Optical sampling depth in the spatial frequency domain

**DOI:** 10.1117/1.JBO.24.7.071603

**Published:** 2018-09-14

**Authors:** Carole K. Hayakawa, Kavon Karrobi, Vivian Pera, Darren Roblyer, Vasan Venugopalan

**Affiliations:** a University of California at Irvine, Department of Chemical Engineering and Materials Science, Irvine, California, United States; b University of California at Irvine, Beckman Laser Institute, Laser Microbeam and Medical Program, Irvine, California, United States; c Boston University, Department of Biomedical Engineering, Boston, Massachusetts, United States

**Keywords:** spatial frequency domain, Monte Carlo simulation, photon migration, diffuse optics, diffuse optical spectroscopy

## Abstract

We present a Monte Carlo (MC) method to determine depth-dependent probability distributions of photon visitation and detection for optical reflectance measurements performed in the spatial frequency domain (SFD). These distributions are formed using an MC simulation for radiative transport that utilizes a photon packet weighting procedure consistent with the two-dimensional spatial Fourier transform of the radiative transport equation. This method enables the development of quantitative metrics for SFD optical sampling depth in layered tissue and its dependence on both tissue optical properties and spatial frequency. We validate the computed depth-dependent probability distributions using SFD measurements in a layered phantom system with a highly scattering top layer of variable thickness supported by a highly absorbing base layer. We utilize our method to establish the spatial frequency-dependent optical sampling depth for a number of tissue types and also provide a general tool to determine such depths for tissues of arbitrary optical properties.

## 1 Introduction

The use of spatial frequency domain (SFD) methods for diffuse optical imaging of biological tissues has gained significant traction in the biophotonics community since its introduction in 1998.^1^ SFD methods combine a measurement of spatially modulated reflectance at multiple spatial frequencies with light transport models to determine optical and physiological properties of the tissue in question. The power of SFD methods is demonstrated most notably in spatial frequency domain imaging (SFDI), where such measurements are made for every pixel in a wide-field image. Such images provide functional mappings of optical and physiological properties with submillimeter detail^2^ and temporal resolution limited to the spatial pattern projection times.^3^

Diffuse optical methods utilize measurements made under multiple illumination/detection configurations whether it be multiple source–detector separations, delay times, and/or spatial/temporal modulation frequencies. These configurations inherently collect photons that have penetrated different tissue volumes. Understanding the spatial regions that detected photons have sampled, and their sensitivity to source–detector configuration, is crucial in many situations, including the assignment of optical properties to a given tissue volume, accounting for the effects of tissue heterogeneities, measuring layered tissues, and the performance of image reconstruction. While this problem has been extensively studied for spatially/temporally resolved and temporal frequency domain methods,^4^^–^^16^ extensive quantitative assessments of the optical sampling depths relevant to SFD methods are not prevalent in the literature.

Knowledge of tissue depths sampled by SFDI is important for understanding and contextualizing measurements of layered and heterogeneous tissue, and is relevant to all clinical and preclinical applications of SFDI described in the literature to date. For example, SFDI has been investigated for multiple applications in human skin, including burns,^17^ reconstructive skin flaps,^18^ and skin malignancies.^19^ The layered structure of skin, which includes the largely avascular superficial epidermis and deeper vascularized papillary and reticular dermis and subcutaneous adipose tissue, requires knowledge of which layers are being probed in order to avoid misleading or irrelevant measurements. For example, in applications involving skin burns, the thickness of affected tissue dictates the treatment protocol, highlighting the importance of understanding the depth of tissue probed.^20^ SFDI is also being explored for deeper tissue applications, including the measurement of human breast tumors,^21^ where it is essential to understand the penetration of collected photons in order to evaluate the maximum depth of measurable tumor contrast. There has been a growing interest in subdiffusive SFDI, which makes use of high spatial frequencies ($>0.2\;{\mathrm{mm}}^{-1}$) and has been used in applications such as tumor margin detection.^22^ Again, quantitation of SFD sampling depth at these higher frequencies will allow users of this technique to determine the probed tissue thickness of resected specimens, which is relevant for interpretation of such results relative to the specific guidelines for clear margins for different tumor types.^23^ In the preclinical setting, SFDI has been used for small animal tumor imaging to better understand cancer treatment response and resistance.^24^ Photon sampling depth is essential for understanding what portion of the collected signal is due to superficial skin versus subcutaneous tumor. Finally, multimodality imaging, in which SFDI is combined with optical sectioning techniques such as multiphoton microscopy or optical coherence tomography, would benefit from knowledge of SFD photon sampling depth to ensure data from each modality is sampling similar tissue volumes.^25^

Prior efforts to analyze SFD sampling depth utilized an approximate solution to the radiative transport equation (RTE) based on a spherical harmonic expansion^26^ with errors dependent upon the order of the expansion. Here, we formulate a method to analyze SFD sampling depth by developing a Monte Carlo (MC) random variable that can be rigorously derived from the RTE. In this method, the error associated with the SFD sampling depth estimates is solely dependent on the number of photon packets launched. We provide both experimental validation for these results and sampling depth statistics for a range of optical properties in a manner that allows for rapid and accurate estimation for any candidate tissue.

We describe four steps toward the use of MC simulations to estimate SFD sampling depths. First, we provide a method to determine optical sampling depth statistics for SFD measurements using MC simulations. Second, we validate our simulated results by performing a series of experimental measurements on fabricated phantoms. Third, we apply our method to determine sampling depths for real tissue types. Finally, we provide tabular data and MATLAB code to generate sampling depth results for any tissue type. The archived version of the code can be freely accessed and executed through Code Ocean: https://codeocean.com/capsule/97500821-e8c9-456c-9a41-3926677bfc79/code.

## 2 Methods

We analyze the sampling depth of SFD measurements using MC simulations that determine the sampling probability of detected photon packets for specific depths. For this analysis, we utilize the modified shortcut method (MSM)^27^ that directly performs an MC simulation of the two-dimensional (2-D) spatial Fourier transform of the RTE. Thus, the MC simulations are performed directly in the SFD and not subject to inaccuracies that can result from computation of the discrete Fourier transform of spatially resolved reflectance simulation data. Our computational model utilizes a representation that segments the tissue into z-plane surfaces at uniform depth increments and determines the subset of detected photon packets that traverse each depth. While the computational system that we consider is spatially homogeneous, the approach that we introduce is general and applicable to any layered tissue geometry.

To validate the computational predictions, we utilize a two-layer phantom system with a highly scattering layer of water, nigrosin, and ${\mathrm{TiO}}_{2}$ particles placed over a highly absorbing layer of nigrosin-doped agar gel. We provide results of SFD measurements performed on this two-layer phantom system with differing thicknesses of the top layer for comparison with the MC simulation results.

Below, we establish a rigorous metric for depth-dependent probability of photon visitation and detection $P_{V\cap D}(z)$ and the use of an MC simulation for its computation. This depth-dependent probability distribution is then related directly to the measured SFD reflectance and used to define various metrics for SFD optical sampling depth. We then discuss the details of our SFD validation measurements.

### 2.1 Monte Carlo Probability of Visitation and Detection $P_{V\cap D}$

We wish to characterize the spatial distribution of only those photons that are launched by a specified source and subsequently captured by a detector of interest. We accomplish this by computing a “contributon” response function that was first developed in the nuclear engineering community for the solution of deep-penetration nuclear transmission problems using MC methods.^28^^–^^34^ In the transmission problems, a surface between the source and detector is first specified. A forward simulation for photon transport from the source is then matched with an adjoint simulation of photon transport from the detector over the midway surface using the contributon response function. In a reflectance geometry, we use this idea to determine the probability that detected photon packets have visited a depth d within the tissue by defining the surface at the selected depth as our midway surface. We determine the probability that photon packets from the source “visit” d, P(V), and then determine the probability that they will subsequently be detected, P(D|V). Bayes theorem^35^ is used to determine the probability of visitation and detection, $P_{V\cap D}$

$$P_{V\cap D}=P(V)P(D|V).$$ (1)

One method to determine only those photon packets that originate from the source, travel to the midway surface, and subsequently arrive at the detector, would be to match the radiance determined by a forward simulation from the source with an adjoint simulation from the detector over the midway surface. This approach works well when the source and detector are “small” relative to the midway surface.^14^^,^^36^ In the biomedical optics community, the use of such coupled forward-adjoint simulations has been used to address fluorescence excitation and detection,^37^ “photon hitting density” maps,^6^ and tomographic sensitivity analysis for spatially resolved reflectance.^14^^,^^36^^,^^38^

**Fig. 1:**
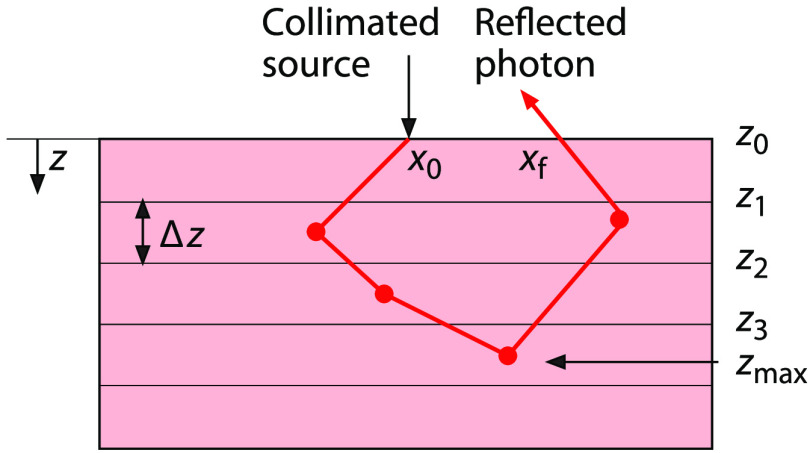
Schematic of an SFD $P_{V\cap D}$ MC simulation within a tissue subdivided into layered surfaces.

In this work, we produce $P_{V\cap D}$ distributions for SFD measurements using a single conventional MC simulation. We take this approach because MC simulations of SFD methods utilize a “small” source and “large” detector, i.e., light is injected into the medium at a single point while detection occurs at all locations on the tissue surface. In such a scenario, a conventional MC approach provides better computational efficiency relative to coupled forward-adjoint methods.^38^ Note that the SFD situation is unique relative to other diffuse optical methods where both “small” sources and “small” detectors are typically used. Specifically, we create depth-dependent SFD $P_{V\cap D}(z)$ distributions by defining parallel x−y planes, placed at regular intervals of 0.01 mm below the tissue surface, as our midway surfaces. We choose this interval size to provide submillimeter resolution for our probability distributions. We then perform an MC simulation that tracks those photon packets that both visit these depths and are subsequently detected. Figure 1 shows a schematic of the tissue segmented into layers using planes located at depths $z_{0}=0,z_{1},z_{2},\cdots$ separated by height Δz. These planes define midway surfaces at depths $z_{i}$ at which $P_{V\cap D}(z_{i})$ is determined. We calculate $P_{V\cap D}(z)$ by tracking each photon packet from the source to these various midway surfaces and from those surfaces to the detector. In doing so, these calculations inherently combine the probability of a photon packet visiting the midway surface after being launched by the source with the probability of detection after visiting the midway surface.

The $P_{V\cap D}(z_{i})$ tallies are computed using an MC simulation of radiative transport by determining the midway surfaces visited by each detected photon packet. As mentioned earlier, we utilize an MC simulation using the MSM^27^ that tallies the photon packet weights directly in the SFD. A narrow, collimated beam normally incident on the tissue surface is used as the source within the simulation and the photon packets are transported using conventional MC propagation.^39^^,^^40^ The complex weights specified by the MSM for the specified spatial frequency provide both the amplitude change and phase shifts that result in photon packet propagation from source to detector. When utilizing illumination modulated along the x-axis at spatial frequency $f_{x}$, the SFD complex tally is^27^

$$\xi =W\;\exp[-2\pi if_{x}(x_{f}-x_{0})],$$ (2)

where ξ is a random variable that represents the tally or detected weight for each photon packet, W is the weight of an individual photon packet as determined by discrete absorption weighting,^41^ $x_{0}$ is the location along the x-axis where the photon packet enters the tissue sample, and $x_{f}$ is the exiting x-axis location immediately prior to detection. Note that Eq. (2) can be used in layered tissue systems. The optical property changes in each layer will be implicitly captured by W and the location of $x_{f}$.

For each detected photon packet, the SFD complex random variable [Eq. (2)] is tallied for each midway surface that the photon packet crossed and notated as $\xi (z_{i})$. For example, if the photon packet reached a maximum depth $z_{\max}$ prior to detection, the photon packet crossed all midway surfaces residing at depths less than $z_{\max}$. For the photon packet trajectory shown in Fig. 1, the detected photon packet weight is tallied to the midway surface $z_{3}$ as well as all midway surfaces residing at shallower depths because the photon packet also crossed those surfaces. Once N photon packet trajectories are simulated, the expected value of ξ for depth $z_{i}$, $E[\xi (z_{i})]$ produces the probability that the trajectory of a detected photon packet crossed depth $z_{i}$, $P_{V\cap D}(z=z_{i})$

$$P_{V\cap D}(z=z_{i})=E[\xi (z_{i})]=\underset{N\to \infty }{\lim}\frac{1}{N}\overset{N}{\underset{j=1}{\sum }}\xi_{j}(z_{i}),$$ (3)

where N is the number of photon packets launched. By determining $\xi (z_{i})$ for all the $z_{i}$ depths under consideration, we form the depth-dependent probability distribution of photon packet visitation and $\text{detection}=P_{V\cap D}(z)$.

### 2.2 Monte Carlo Maximum Depth of Penetration $P_{z_{\text{max}}}$

The $P_{V\cap D}(z)$ distribution over all depths z does not result in a directly measurable quantity because photon packets that contribute to the $P_{V\cap D}$ tally at a surface $z_{i}$ also contribute to $P_{V\cap D}$ tallies at all locations shallower than $z_{i}$. However, we can use $P_{V\cap D}(z)$ to derive a distribution that isolates the contribution of each detected photon packet to a single bin corresponding to the maximum depth visited by that photon packet trajectory. In doing so, we ensure that each detected photon packet is tallied to only a single depth bin within the tissue. This distribution is formed by taking differences of the $P_{V\cap D}(z)$ tally in successive bins

$$P_{z_{\max}}(z_{i})=P_{V\cap D}(z=z_{i})-P_{V\cap D}(z=z_{i+1}).$$ (4)

We call $P_{z_{\max}}(z)$ the “$z_{\max}$” distribution which isolates those detected photon packets that crossed into depth $z_{i}$ but did not cross into $z_{i+1}$. Because each photon packet is tallied only once at its maximum depth of propagation “$z_{\max}$,” $P_{z_{\max}}(z)$ has the property that its integral over all depths z recovers all the weight of all the detected photon packets, which is equivalent to the total diffuse reflectance, $R_{d}$

$$R_{d}=\int_{0}^{\infty }P_{z_{\max}}(z)dz.$$ (5)

If the upper limit of integration on the right-hand side of the above equation is taken instead to some finite depth d, the result would tally only those photon packets that contribute to the reflectance and whose trajectories were restricted to depths less than or equal to d. We notate this as $P_{z_{\max}}(z\leq d)$ or

$$P_{z_{\max}}(z\leq d)=\int_{0}^{d}P_{z_{\max}}(z)dz.$$ (6)

This construct will be used to validate our MC simulation results with our SFD measurements.

Division of $P_{z_{\max}}(z\leq d)$ by $R_{d}$ produces a probability distribution function that describes the fraction (X) of the detected light that visited tissue depths d or less

$$X=\frac{P_{z_{\max}}(z\leq d)}{R_{d}}.$$ (7)

By setting the value of X in the above equation to a given value, say 0.5 (or 50%), we can calculate the maximum tissue depth $d_{50}$ from which 50% of the detected reflectance emanates.

### 2.3 Experimental Validation

**Fig. 2:**
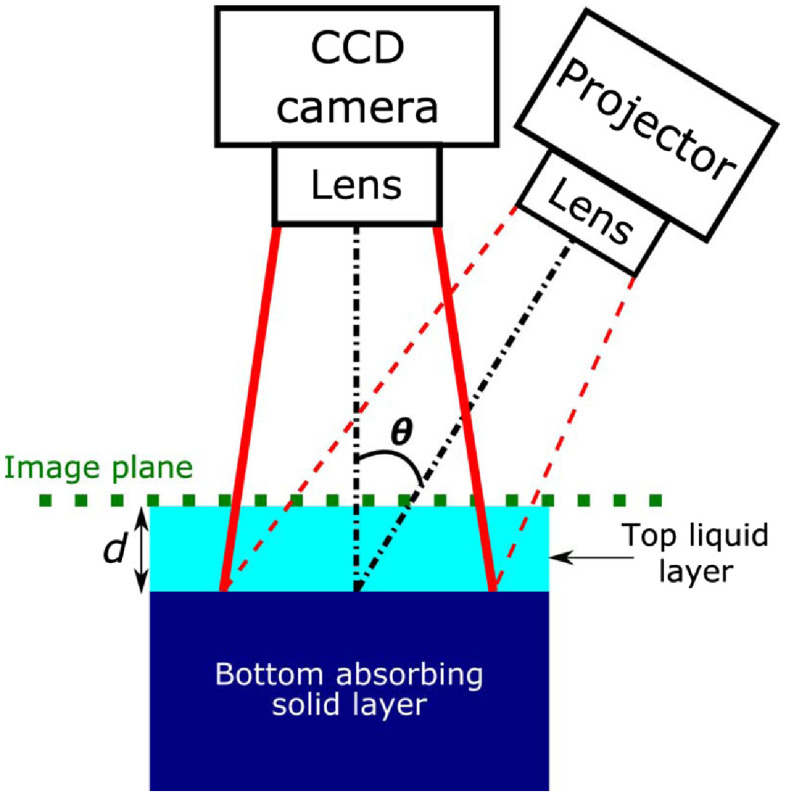
Experimental setup and schematic of two-layer phantom with top layer thickness d varying from $[0-7.5]l^{*}$ where $l^{*}=2\;\mathrm{mm}$. Theta (θ) is 15 deg. This experimental setup was used to acquire reflectance as a function of spatial frequency $\left(f_{x}\right)$ and layer thickness d. All measurements were performed at λ=731  nm.

We used experimental SFD measurements to validate our computational results. These measurements were taken in a two-layer phantom designed to determine the optical sampling depth as a function of spatial frequency. The two-layer phantom was housed in a container with (L×W×H) dimensions of 7.2  cm×10.8  cm×6.1  cm. The top layer of the phantom was a liquid composed of water, nigrosin, and ${\mathrm{TiO}}_{2}$ particles with optical properties $\mu_{s}^{\prime }/\mu_{a}=100$ and $l^{*}=1/(\mu_{a}+\mu_{s}^{\prime })=2\;\mathrm{mm}$ at λ=731  nm. The bottom layer was a highly absorbing solid phantom composed of agar, water, and nigrosin, and occupied a total volume of 350 mL in the container. The top layer thickness d was varied from $[0-7.5]l^{*}$. Figure 2 provides a schematic of the setup.

We used the OxImager RS SFDI system (Modulated Imaging Inc., Irvine, California) to measure the two-layer phantom. The SFDI system utilizes crossed linear polarizers in front of the projection and detection lenses to minimize the effect of specular reflection and select for diffuse reflection. The projection field of view (FOV) was 20  cm×15  cm and directed to the tissue surface at an angle of 15 deg relative to the surface normal. Detection was performed perpendicular to the surface of the phantom with an FOV of 8  cm× 6  cm (effective NA = 0.253). A vertical translation stage was used to support and adjust the height of the container with the two-layer phantom. After taking the first measurement of the highly absorbing solid phantom (i.e., d=0  mm), incremental amounts of the liquid phantom were added successively with a predetermined volume such that the top liquid layer thickness d above the solid phantom increased by 0.5 mm between each measurement following the d=0  mm measurement. The micrometer on the translation stage was used to lower the two-layer phantom system by 0.5 mm following each measurement such that the top surface of the two-layer phantom system remained at a constant image plane for all top layer thicknesses measured. This was done to avoid the need for height correction during data processing. All measurements were performed at a wavelength λ=731  nm with spatial frequencies $f_{x}=0$, 0.0125, 0.025, 0.0375, 0.05, 0.0625, 0.075, 0.0875, 0.1, 0.125, 0.15, 0.175, 0.2, 0.25, and $0.3\;{\mathrm{mm}}^{-1}$. At each spatial frequency (in this case, along one spatial dimension x), raw reflectance images at three different phases (0, 2π/3, and 4π/3 radians) were sequentially projected onto the phantom using a digital micromirror device. The resulting reflected light was imaged with a camera. The images were then demodulated to extract the amplitude envelope for each spatial frequency measurement using an established amplitude demodulation algorithm.^42^^,^^43^ A separate reference measurement at the same spatial frequencies was made on a calibration phantom with known optical properties for calibration of the SFDI source intensity and instrument response. This calibration enables the measured reflectance to be converted to absolute reflectance. This is achieved by comparing the measured reflectance from the calibration phantom with its predicted diffuse reflectance from an MC-based forward model using the phantom’s known optical properties. The region of interest (ROI) chosen for data analysis was centered in the detection FOV to avoid edge effects and measured 7.5  cm×2  cm. The reported experimental data are average values taken over the ROI.

## 3 Results and Discussion

We first present $P_{V\cap D}(z)$ results from which we compute $P_{z_{\max}}(z)$. The $P_{z_{\max}}(z)$ results will form the basis for (a) validating our computational method with experimental measurements and (b) determining metrics for optical sampling depth. We then present optical sampling depth results for a variety of tissue types based on literature reported optical properties.

### 3.1 Probability of Visitation and Detection $P_{V\cap D}$

We first consider a highly scattering tissue system with refractive index n=1.4 with optical properties providing $(\mu_{s}^{\prime }/\mu_{a})=100$ and $l^{*}=1\;\mathrm{mm}$ and single-scattering anisotropy g=0.8. This corresponds to optical absorption and scattering coefficients $\mu_{a}=0.00990099\;{\mathrm{mm}}^{-1}$ and $\mu_{s}=4.95049505\;{\mathrm{mm}}^{-1}$, respectively. In the MC simulation, we utilize a narrow collimated beam normally incident into the tissue. Using the method described in Sec. 2.1, we launched $N=10^{8}$ photon packets to obtain $P_{V\cap D}(z)$ with z bins incremented at 0.01 mm and a set of spatial frequencies spanning 0 and $0.5\;{\mathrm{mm}}^{-1}$.

**Fig. 3:**
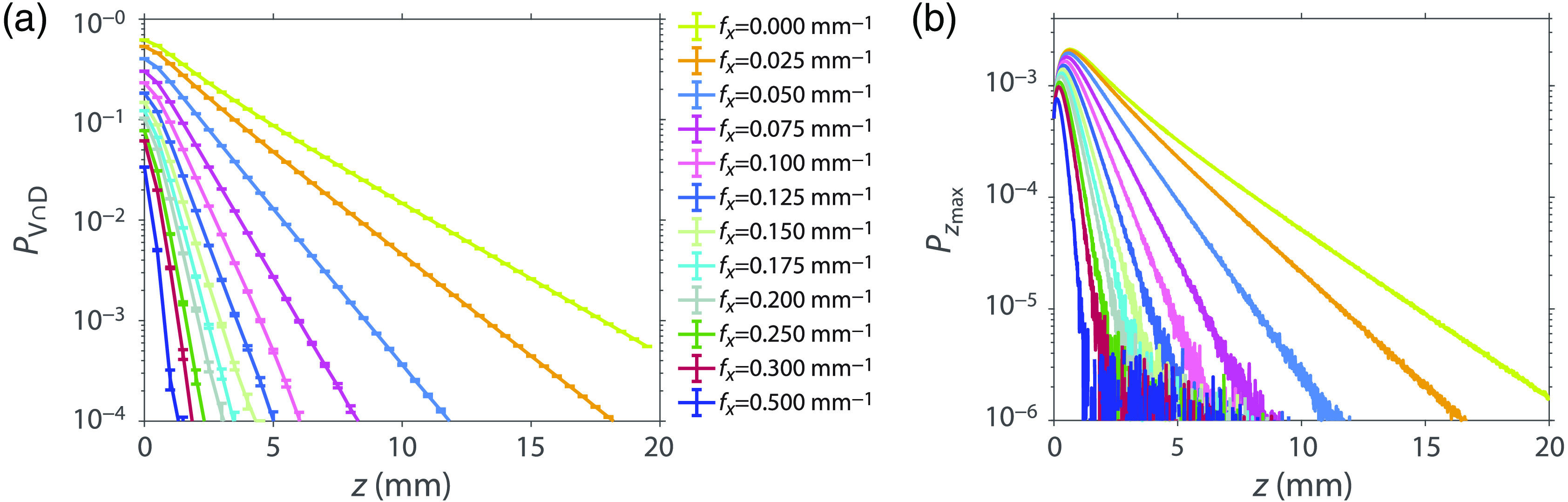
(a) $P_{V\cap D}(z)$ generated for media with optical properties $\mu_{s}^{\prime }/\mu_{a}=100$, $l^{*}=1\;\mathrm{mm}$ using $f_{x}=0,0.025,0.05,0.075,0.1,0.125,0.15,0.175,0.2,0.250,0.3,0.5\;{\mathrm{mm}}^{-1}$ with 1−σ error bars. Data were obtained at a Δz=0.01  mm. Data symbols are shown on this plot at z-intervals of 1 mm. (b) $P_{z_{\max}}(z)$ derived from $P_{V\cap D}(z)$ distributions shown in (a).

Figure 3(a) shows the $P_{V\cap D}(z)$ distributions obtained for spatial frequencies $f_{x}=0$, 0.025, 0.05, 0.075, 0.1, 0.125, 0.15, 0.175, 0.2, 0.250, 0.3, and $0.5\;mm^{-1}$. The value of the $P_{V\cap D}$ at the first surface z=0  mm, $P_{V\cap D}(z=0)$, is equivalent to the total diffuse reflectance for each spatial frequency, $R_{d}(f_{x})$. This is because every diffusely reflected photon packet will traverse this surface. The plots are shown with 1−σ error bars which indicate that 68% of independent simulations will produce results, which lie within this interval. The $f_{x}=0\;{\mathrm{mm}}^{-1}$ plot has a value of 0.62 at z=0  mm representing total diffuse reflectance and decays to $4.7\times 10^{-4}$ by depth z=20  mm, representing approximately a 3 order of magnitude reduction. The $f_{x}=0.5\;{\mathrm{mm}}^{-1}$ plot has a value of 0.03 at z=0 and decays to $3.6\times 10^{-7}$ at z=20  mm, nearly a 6 order of magnitude reduction. These data illustrate the low-pass optical transport characteristics of scattering tissues.

Figure 3(b) shows the $P_{z_{\max}}(z)$ derived from $P_{V\cap D}(z)$ using Eq. (4). As described in Sec. 2.2, the benefit of transforming the $P_{V\cap D}(z)$ results to the max $z_{i}$ formulation $P_{z_{\max}}(z=z_{i})$ is that (a) $P_{z_{\max}}(z=z_{i})$, when integrated over all $z_{i}$, produces total diffuse reflectance $R_{d}$ and (b) it effectively tracks the maximum depth sampled by each photon packet and provides group statistics on sampling depth for each spatial frequency.

### 3.2 Experimental Validation

The experimental setup described in Sec. 2.3 consists of measurements taken from a two-layer phantom with a highly absorbing bottom layer placed at various depths d that extinguishes any photons that propagate to that depth. The resulting measured reflectance is composed of only photons that never reach depths z>d, i.e., the photons detected possess trajectories with a maximum z≤d. The analogous computational result is given by Eq. (6).

**Fig. 4:**
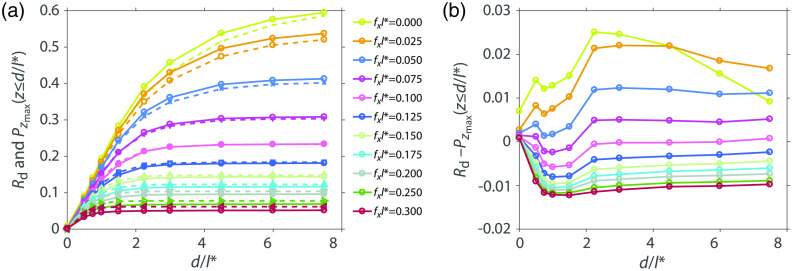
(a) Experimentally measured and calibrated $R_{d}$ (solid lines) and $P_{z_{\max}}(z\leq d/l^{*})$ from simulation (dashed lines) and (b) their difference.

Figure 4(a) shows the calibrated experimental measurements of diffuse reflectance versus top layer thickness d and plots of $P_{z_{\max}}(z\leq d)$ and (b) their difference. The plot shows a subset of the measured $f_{x}$ values for clarity. The depths d and the spatial frequencies have been normalized to $l^{*}=1\;\mathrm{mm}$. For normalized spatial frequency $f_{x}l^{*}=0$, the plot rises from 0 and reaches a value of 0.595 for top layer thickness of $7.5\;d/l^{*}$. The plot rises monotonically as the top layer thickness increases because increasing numbers of photons fail to be extinguished by the bottom layer and are able to return to the surface to contribute to reflectance. As the spatial frequency increases, the measured diffuse reflectance flattens at a certain depth indicating that spatially modulated light for this frequency does not interrogate the tissue below that depth. For example, for $f_{x}l^{*}=0.3$, the spatially modulated reflectance rises from 0 and rises to 0.05 for top layer thickness of $1.0\;d/l^{*}$ without further increases for larger top layer thicknesses. The absolute difference between the experimental measurements and the computational predictions ranges between [−0.012,0.025].

### 3.3 Metrics for Optical Penetration Depth

**Fig. 5:**
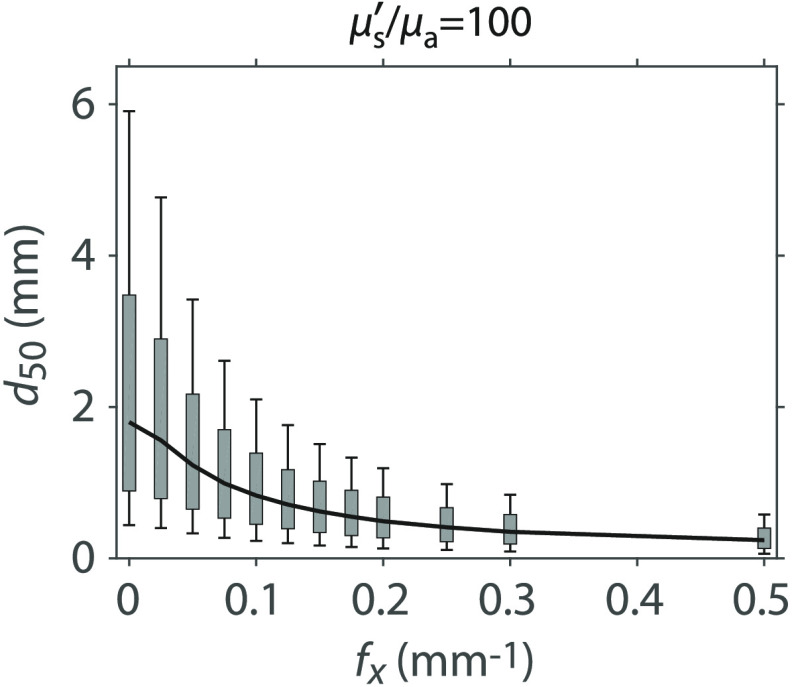
Median sampling depth ($d_{50}$) with [25 to 75]% (gray rectangle) and [10 to 90]% (vertical-capped line) intervals versus $f_{x}$ for media with optical properties $\mu_{s}^{\prime }/\mu_{a}=100$ and $l^{*}=1\;\mathrm{mm}$. Table of plot values are provided in Table 3.

We can determine the depth-dependent variation of the detected photon packets by determining the tissue depth that contains the complete photon packet trajectories corresponding to a certain fraction of the total measured diffuse reflectance at a given spatial frequency. For example, the spatial frequency dependence of sampling depth that encloses all the photon packet trajectories corresponding to only 50% of the detected reflectance can be determined by finding the value $d_{50}$ that results in a value of X=0.5 using Eq. (7). Similarly, by substituting alternate values for X=0.1, 0.25, 0.75, and 0.9 into Eq. (7), we computed depths $d_{10}$, $d_{25}$, $d_{75}$, and $d_{90}$ that correspond to the tissue depths that enclose the photon packet trajectories responsible for 10%, 25%, 75%, and 90% of the detected reflectance, respectively. These are shown in Fig. 5 with specific numerical values provided in Table 3. The span of the gray rectangles [25 to 75]% and vertical-capped lines [10 to 90]% provides range of depths sampled by these portions of the detected reflectance for these optical properties. In Appendix B, we provide similar plots and tables for a several $\mu_{s}^{\prime }/\mu_{a}$ values to show how these spans vary with optical properties.

These depth metrics are determined from the $z_{\max}$ distribution and provide a direct correspondence between the maximum tissue depths sampled by portions of the detected reflectance. Unlike the photon hitting density,^6^ these depths do not tally the pathlengths that the detected photon packet traverses within the tissue volumes considered and do not map directly to absorption sensitivity. Rather they provide tissue depths that correspond to portions of the detected reflectance. These allow the SFD user to determine what fraction of the measured reflectance is available to interact with the tissue beyond a certain depth. For example, if a given tissue has a $d_{90}=5\;\mathrm{mm}$, the user is certain that 90% of the measured reflectance is restricted to tissue depth ≤5  mm whereas only 10% of the measured reflectance has the opportunity to sample tissue depth >5  mm.

### 3.4 Sampling Depth Estimates for Real Tissue Types

**Fig. 6:**
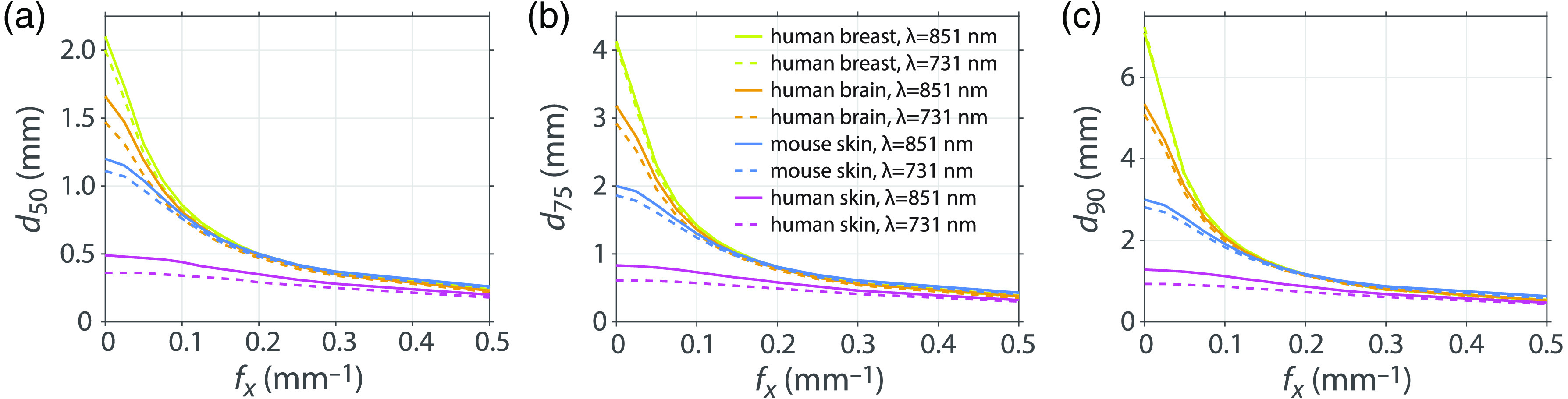
(a) $d_{50}$, (b) $d_{75}$, (c) $d_{90}$ sampling depths for human brain, mouse skin, human skin and human breast tissues at λ=851  nm (solid lines) and 731 nm (dashed lines).

The results provided in Fig. 5 can be generated for any set of optical properties. We apply our methodology to various tissue types to determine the depths that correspond to detection of 50%, 75%, and 90% of the total reflectance at a given spatial frequency. Table 1 shows the tissue types and wavelengths considered and the corresponding optical properties.^44^^–^^47^ We performed independent MC simulations for each tissue type and wavelength pair to determine depth estimates for each. Figure 6 shows the predicted sampling depths versus spatial frequency for the various pairs of tissue type and wavelength considered.

**Table 1:** Tissue optical properties^44^^–^^47^ used in our sampling depth analysis.

Tissue type	λ (nm)	$\mu_{a}({\mathrm{mm}}^{-1})$	$\mu_{s}({\mathrm{mm}}^{-1})$	g	$l^{*}$	n	$\mu_{s}^{\prime }/\mu_{a}$	$\mu_{s}^{\prime }({\mathrm{mm}}^{-1})$
Human breast	731	0.0044	37.51	0.97	0.89	1.4728	255.77	1.13
Human breast	851	0.0066	31.95	0.97	1.04	1.4728	145.24	0.96
Human brain	731	0.0090	16.41	0.92	0.76	1.4026	145.83	1.31
Human brain	851	0.0124	12.84	0.92	0.96	1.4026	82.85	1.03
Mouse skin	731	0.0937	7.73	0.9	1.15	1.4	8.25	0.77
Mouse skin	851	0.1070	6.17	0.9	1.38	1.4	5.77	0.62
Human skin	731	0.2437	16.32	0.8357	0.34	1.4637	11.00	2.68
Human skin	851	0.1563	16.71	0.8707	0.43	1.4637	13.82	2.16

The tissue type, in which spatially modulated illumination can probe most deeply at the smaller spatial frequencies, is human breast at λ=851 and 731 nm. This is due to the higher $\mu_{s}^{\prime }/\mu_{a}$ properties for this tissue, 256 and 145, respectively, along with moderate transport mean-free path values of $l^{*}=0.89\;\mathrm{mm}$ and 1.04 mm, respectively. The median sampling depth of the human brain is 20% smaller than for human breast. While human brain tissue at λ=731  nm has the equivalent $\mu_{s}^{\prime }/\mu_{a}$ value as human breast at λ=851  nm, the transport mean-free path in human brain is only $l^{*}=0.76\;\mathrm{mm}$ as compared to $l^{*}=1.04\;\mathrm{mm}$ in human breast. While mouse skin has the lowest $\mu_{s}^{\prime }/\mu_{a}$ values of the four tissue types considered, which would suggest more superficial optical sampling depths, the transport mean-free paths are the largest of the tissues considered resulting in optical sampling depths that are only slightly more superficial than human brain tissue at low spatial frequencies. The $\mu_{s}^{\prime }/\mu_{a}$ values for human skin are not much larger than mouse skin but with much higher scattering properties resulting in the smallest $l^{*}$ values of the group and the most superficial optical sampling depths.

The spatial frequency dependence characteristics of these optical sampling depths are also of interest. For spatial frequencies larger than $f_{x}=0.1\;{\mathrm{mm}}^{-1}$, differences in the optical sampling in human breast, human brain, and mouse skin are barely distinguishable. For human brain, the median depth values for $f_{x}=0.1\;{\mathrm{mm}}^{-1}$ are roughly half the median depth values using $f_{x}=0\;{\mathrm{mm}}^{-1}$ for both wavelengths. For mouse skin, the median depths at $f_{x}=0.1\;{\mathrm{mm}}^{-1}$ are about two-thirds the median depth values for $f_{x}=0\;{\mathrm{mm}}^{-1}$ for both wavelengths. The lower $\mu_{s}^{\prime }/\mu_{a}$ properties of both human skin and mouse skin are indicative of a diminished effect of scattering on the light transport and result in far less spatial frequency variation in the optical sampling depth. For human skin, the median depth at $f_{x}=0.1\;{\mathrm{mm}}^{-1}$ is 94% of the median depth value for $f_{x}=0\;{\mathrm{mm}}^{-1}$ using λ=731  nm and this factor is 90% at λ=851  nm. For mouse skin, the median depth values at $f_{x}=0.1\;{\mathrm{mm}}^{-1}$ are about 82% the median depth values for $f_{x}=0\;{\mathrm{mm}}^{-1}$ for both wavelengths. At $f_{x}=0.5\;{\mathrm{mm}}^{-1}$, the median depth for all real tissue types is within the range [0.15 to 0.26] mm.

### 3.5 Determination of Optical Sampling Depths for Other Tissue Types

**Fig. 7:**
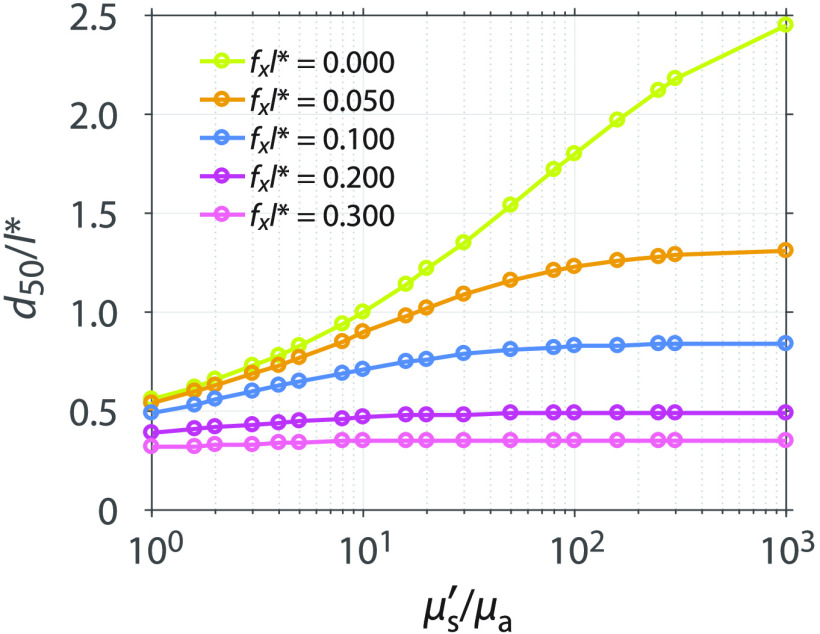
Median sampling depth $d_{50}$ normalized by the transport mean-free path $l^{*}$ as a function of $\mu_{s}^{\prime }/\mu_{a}$ at spatial frequencies $f_{x}l^{*}=0$, 0.05, 0.1, 0.2, and 0.3. For clarity, results for only a subset of $f_{x}$ values are plotted here. Results for additional $f_{x}$ values are provided in the supplemental data.

To enable the determination of optical sampling depths for any tissue type, we used our methodology to determine the variation of optical sampling depth with spatial frequency for a range of $\mu_{s}^{\prime }/\mu_{a}$ values between 1 and 1000 while keeping $l^{*}=1\;\mathrm{mm}$ fixed and assuming g=0.8 and n=1.4. The specific optical properties considered are listed in Table 2. Figure 7 shows the median depth of optical sampling as a function of $\mu_{s}^{\prime }/\mu_{a}$. In this figure, both the median sampling depth and the spatial frequency of illumination are scaled relative to $l^{*}$. The plots show that as the $\mu_{s}^{\prime }/\mu_{a}$ increases, so does the median depth. Moreover, at larger spatial frequencies, there is less sensitivity of the sampling depth to variations in $\mu_{s}^{\prime }/\mu_{a}$.

**Table 2:** General optical properties with $l^{*}=1\;\mathrm{mm}$ used for our sampling depth lookup table.

$\mu_{s}^{\prime }/\mu_{a}$	$\mu_{a}({\mathrm{mm}}^{-1})$	$\mu_{s}^{\prime }{(\mathrm{mm}}^{-1})$	$\mu_{s}^{\prime }/\mu_{a}$	$\mu_{a}{(\mathrm{mm}}^{-1})$	$\mu_{s}^{\prime }{(\mathrm{mm}}^{-1})$
1	0.5	0.5	20	0.04761904	0.95238095
1.6	0.38461539	0.61538462	30	0.03225807	0.96774194
2	0.33333333	0.66666666	50	0.01960784	0.98039216
3	0.25	0.75	80	0.01234568	0.98765432
4	0.2	0.8	100	0.00990099	0.99009901
5	0.16666667	0.83333333	160	0.00621118	0.99378882
8	0.11111111	0.88888889	250	0.00398406	0.99601593
10	0.09090909	0.90909091	300	0.00332226	0.99667774
16	0.05882353	0.94117647	1000	0.00099900	0.99900000

**Fig. 8:**
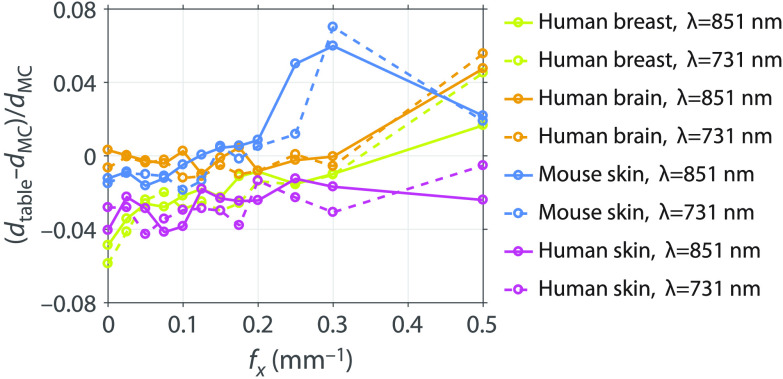
Relative difference between the median depth determined by the lookup table of general optical properties and the median depth determined by running an MC simulation using the real tissue optical properties.

Optical sampling depths determined using the general optical properties can be composed into a lookup table, then scaled, and interpolated to provide depth statistics for an arbitrary tissue. Only knowledge of the tissue absorption and reduced scattering properties is needed. The details of how this is performed are given in Appendix B. The advantage of this lookup table method is that it dispenses with the need to execute an MC simulation for the specific tissue optical properties in question and enables rapid estimation of SFD sampling depths. We performed the lookup table method using the $\mu_{a}$ and $\mu_{s}^{\prime }$ values of the real tissue optical properties listed in Table 1. Figure 8 shows the relative differences between the median depth $d_{\mathrm{table}}$ determined by the lookup table method and the median depth determined by the independent MC simulation at the real tissue optical properties $d_{\mathrm{MC}}$, $(d_{\text{table}}-d_{\mathrm{MC}})/d_{\mathrm{MC}}$. The lookup table estimates and the independent MC simulation results agree to within 7%, suggesting that the lookup table provides an accurate and convenient method for determining depth predictions.

## 4 Conclusions and Future Work

We have presented a transport-rigorous MC method to determine optical sampling depth statistics in the SFD. This method provides depth-dependent probability distributions of photon visitation and detection [$P_{V\cap D}(z)$] for each spatial frequency within homogeneous or layered tissue. Our sampling depth predictions were validated experimentally using SFD measurements taken on a custom fabricated two-layer phantom system. Excellent agreement was obtained between these measurements and our MC predictions.

We applied our method to provide depth sampling statistics for a variety of tissue types at commonly used wavelengths. We nondimensionalized our results to create a 2-D lookup table to determine sampling depth statistics for any tissue given knowledge of the absorption and reduced scattering properties of the tissue. We provide this table and associated computer code to enable its use in the supplemental material.

Collectively, this work provides a rigorous methodology and convenient means to determine optical sampling depth in the SFD. Moving forward, we wish to analyze the effect of spatial-frequency-dependent variations in optical sampling depth on the extraction of optical properties when using SFD measurements at two or more spatial frequencies.^43^ The use of SFD measurements at multiple spatial frequencies results in differential penetration depths leading to a partial volume effect for measurements taken in heterogeneous media. This effect is potentially reduced by choosing spatial frequencies proximal to each other, but this can compromise the ability to accurately extract optical properties.^48^ Future work will aim to evaluate the trade-offs between partial volume effects and optical property extraction errors when choosing spatial frequencies for specific applications.

## Figures and Tables

**Fig. 9:**
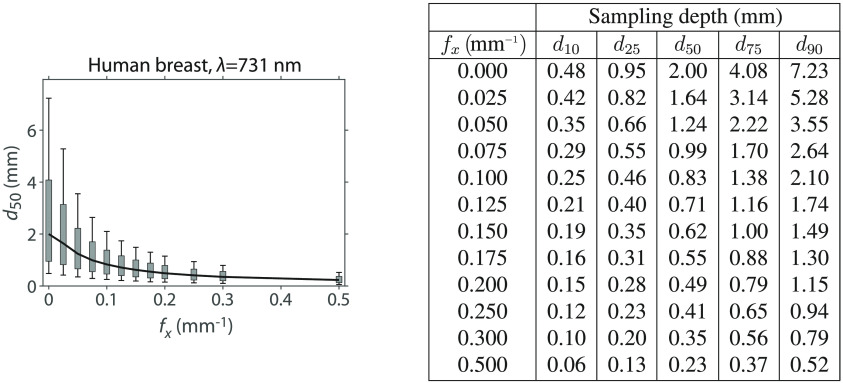
Median sampling depth ($d_{50}$) with [25 to 75]% (gray rectangle) and [10 to 90]% (vertical-capped line) intervals versus $f_{x}$ and table of plot values for human breast at 731 nm.

**Fig. 10:**
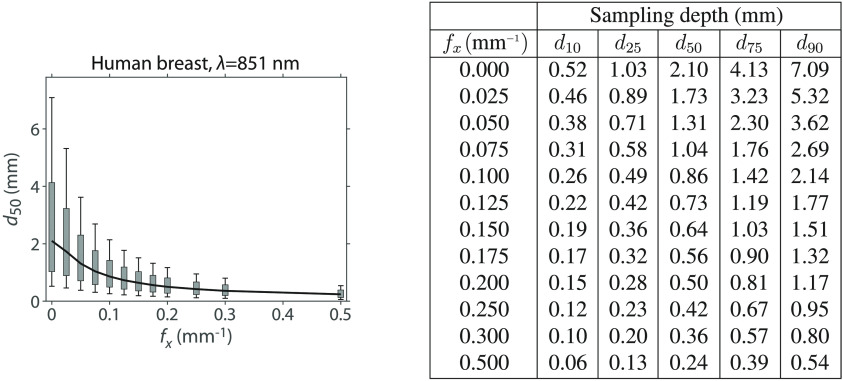
Median sampling depth ($d_{50}$) with [25 to 75]% (gray rectangle) and [10 to 90]% (vertical-capped line) intervals versus $f_{x}$ and table of plot values for human breast at 851 nm.

**Fig. 11:**
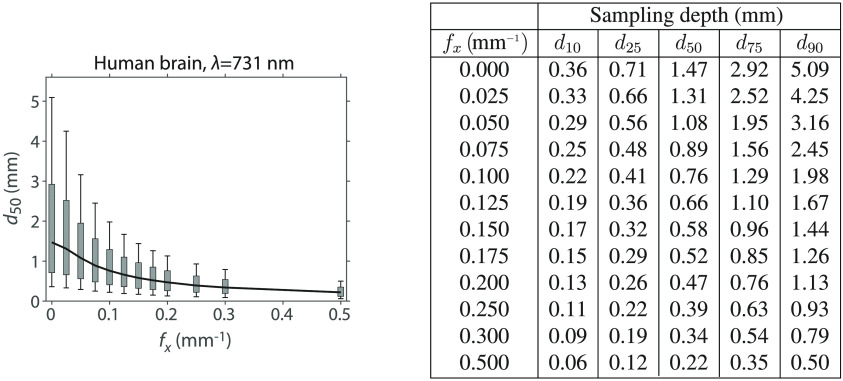
Median sampling depth ($d_{50}$) with [25 to 75]% (gray rectangle) and [10 to 90]% (vertical-capped line) intervals versus $f_{x}$ and table of plot values for human brain at 731 nm.

**Fig. 12:**
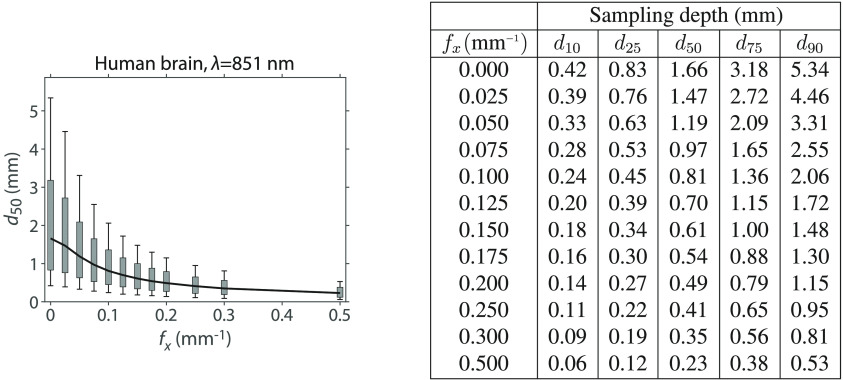
Median sampling depth ($d_{50}$) with [25 to 75]% (gray rectangle) and [10 to 90]% (vertical-capped line) intervals versus $f_{x}$ and table of plot values for human brain at 851 nm.

**Fig. 13:**
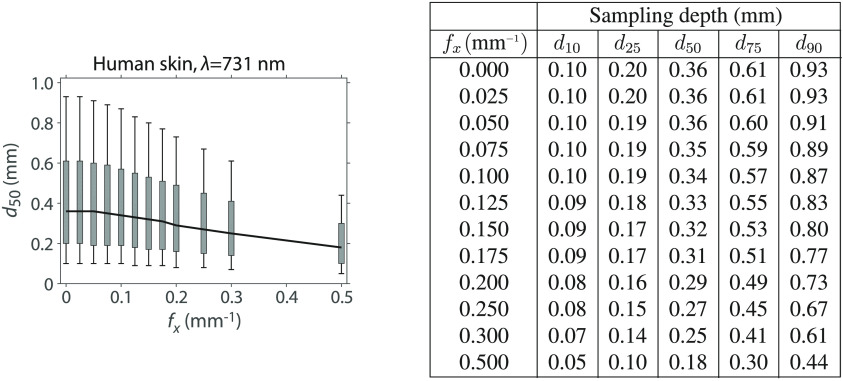
Median sampling depth ($d_{50}$) with [25 to 75]% (gray rectangle) and [10 to 90]% (vertical-capped line) intervals versus $f_{x}$ and table of plot values for human skin at 731 nm.

**Fig. 14:**
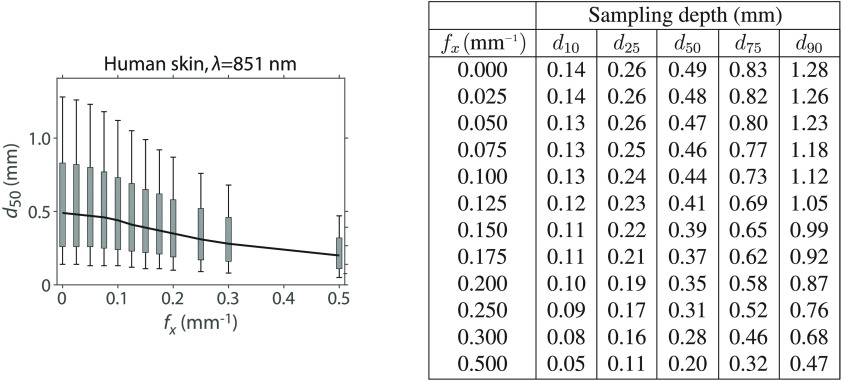
Median sampling depth ($d_{50}$) with [25 to 75]% (gray rectangle) and [10 to 90]% (vertical-capped line) intervals versus $f_{x}$ and table of plot values for human skin at 851 nm.

**Fig. 15:**
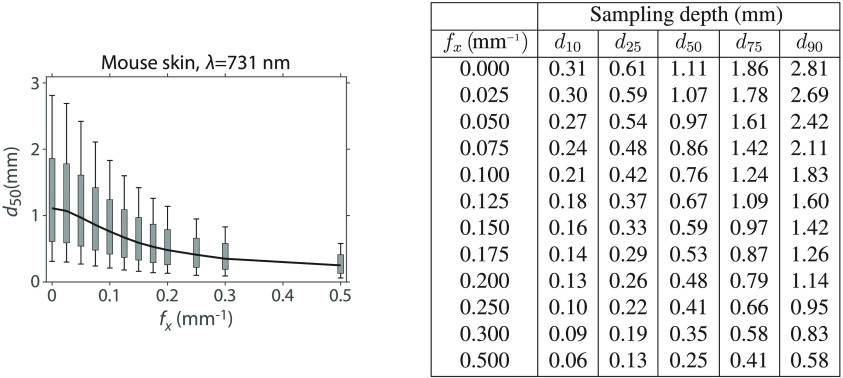
Median sampling depth ($d_{50}$) with [25 to 5]% (gray rectangle) and [10 to 90]% (vertical-capped line) intervals versus $f_{x}$ and table of plot values for mouse skin at 731 nm.

**Fig. 16:**
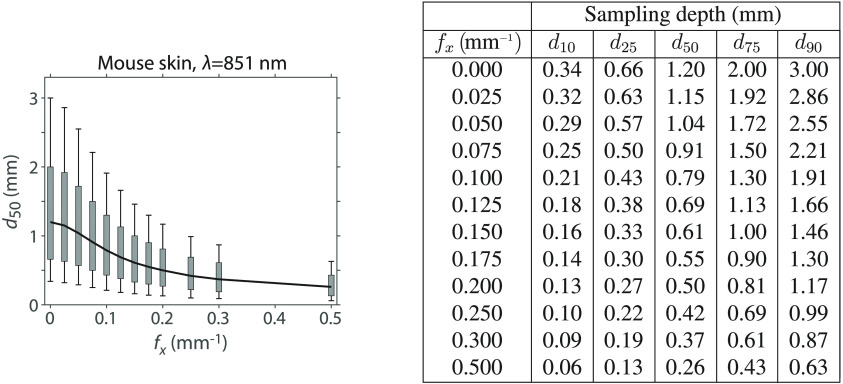
Median sampling depth ($d_{50}$) with [25 to 75]% (gray rectangle) and [10 to 90]% (vertical-capped line) intervals versus $f_{x}$ and table of plot values for mouse skin at 851 nm.

**Fig. 17:**
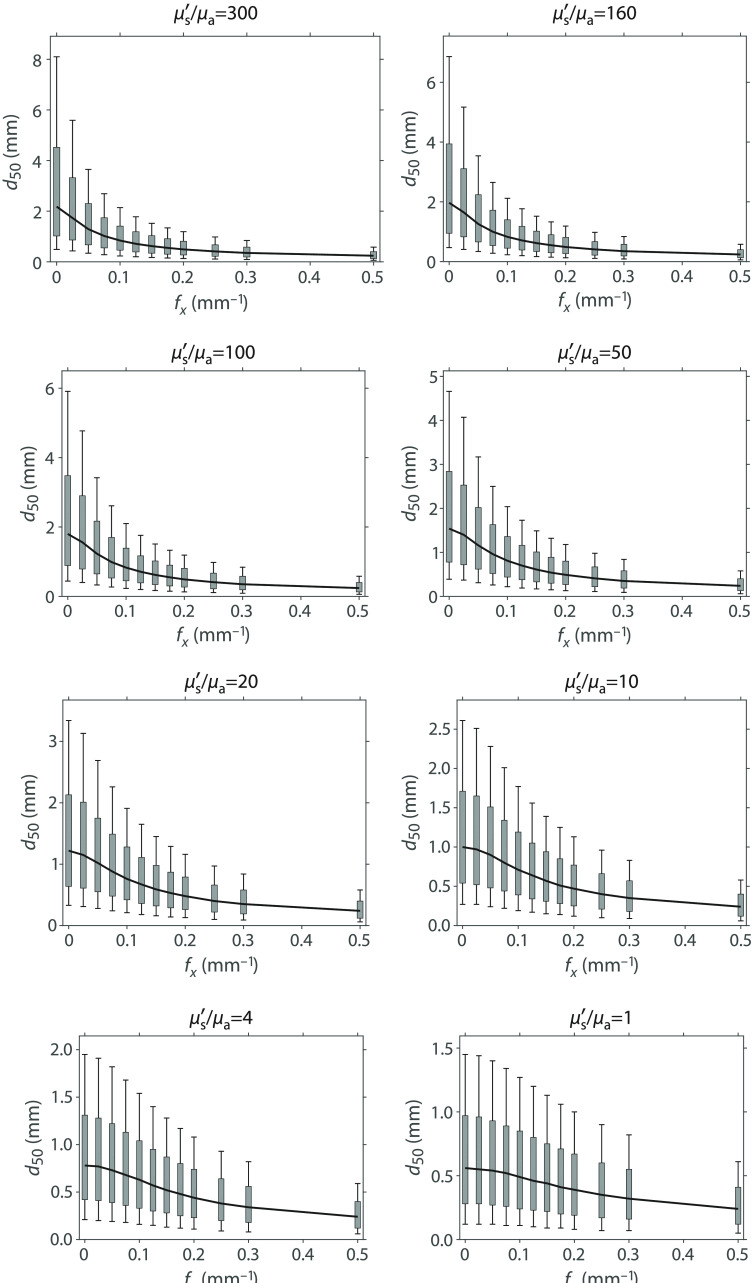
Median sampling depth ($d_{50}$) with [25 to 75]% (gray rectangle) and [10 to 90]% (vertical-capped line) intervals versus $f_{x}$ for general tissue properties. Values of $\mu_{s}^{\prime }/\mu_{a}$ plotted are a subset of those in the supplemental material.

## Appendix A: Real Tissue Data Details

In Figs. 9–16, we provide detailed sampling depth metrics for the specific tissue types and wavelengths as listed in Table 1 with partial results shown in Fig. 6.

## Appendix B: General Tissue Data Supplemental Tables and Code

We generated a table of data for general tissue optical properties that can be used to estimate sampling depths for any arbitrary tissue i given knowledge of the $\mu_{a,i}$ and $\mu_{s,i}^{\prime }$ of the candidate tissue. The data consist of 2-D lookup tables for the 10%, 25%, 50%, 75%, and 90% sampling depths (Table 3). Each table has $\mu_{s}^{\prime }/\mu_{a}$ along one axis and $f_{x}l^{*}$ along the other. The $\mu_{s}^{\prime }/\mu_{a}$ values are those listed in Table 2. The $f_{x}l^{*}$ values are: 0, 0.01, 0.02, 0.025, 0.03, 0.04, 0.05, 0.06, 0.07, 0.075, 0.08, 0.09, 0.1, 0.12, 0.125, 0.14, 0.15, 0.16, 0.175, 0.18, 0.2, 0.25, 0.3, 0.5, and 0.7.

To determine the sampling depth for a candidate tissue with optical properties $\mu_{a}$ and $\mu_{s}^{\prime }$, each table entries and axes are scaled appropriately and then a 2-D linear interpolation method, interp2 (MATLAB 2016b), is used to determine the sampling depth at the spatial frequency of interest. Specifically, to scale the table appropriately, the entries are multiplied by $l^{*}$ of the candidate tissue, and the $f_{x}l^{*}$ axis is divided by $l^{*}$ of the candidate tissue. Then the $\mu_{s}^{\prime }/\mu_{a}$ of the candidate tissue and the spatial frequencies $f_{x}$ of interest are used to interpolate into each table to produce sampling depth estimates.

The tabular data and code to interpolate the data are given in the supplemental material.

**Table 3:** Depth statistics for general tissue properties. Data for $\mu_{s}^{\prime }/\mu_{a}$ values presented are a subset of those in the supplemental material.

$\mu_{s}^{\prime }/\mu_{a}=300$	Sampling depth (mm)				
$f_{x}({\mathrm{mm}}^{-1})$	$d_{10}$	$d_{25}$	$d_{50}$	$d_{75}$	$d_{90}$
0.000	0.49	1.02	2.18	4.52	8.10
0.025	0.43	0.86	1.73	3.32	5.59
0.050	0.34	0.67	1.29	2.30	3.65
0.075	0.28	0.55	1.02	1.74	2.69
0.100	0.23	0.46	0.84	1.41	2.14
0.125	0.20	0.39	0.71	1.19	1.78
0.150	0.17	0.34	0.62	1.03	1.52
0.175	0.15	0.30	0.55	0.91	1.34
0.200	0.13	0.27	0.49	0.81	1.19
0.250	0.11	0.22	0.41	0.67	0.98
0.300	0.09	0.19	0.35	0.58	0.84
0.500	0.06	0.13	0.24	0.40	0.58
$\mu_{s}^{\prime }/\mu_{a}=160$	Sampling depth (mm)				
$f_{x}({\mathrm{mm}}^{-1})$	$d_{10}$	$d_{25}$	$d_{50}$	$d_{75}$	$d_{90}$
0.000	0.47	0.95	1.97	3.94	6.86
0.025	0.41	0.83	1.65	3.11	5.17
0.050	0.34	0.66	1.26	2.24	3.54
0.075	0.28	0.54	1.00	1.72	2.65
0.100	0.23	0.45	0.83	1.40	2.12
0.125	0.20	0.39	0.71	1.18	1.77
0.150	0.17	0.34	0.62	1.02	1.52
0.175	0.15	0.30	0.55	0.90	1.33
0.200	0.13	0.27	0.49	0.81	1.19
0.250	0.11	0.22	0.41	0.67	0.98
0.300	0.09	0.19	0.35	0.58	0.84
0.500	0.06	0.13	0.24	0.40	0.58
$\mu_{s}^{\prime }/\mu_{a}=100$	Sampling depth (mm)				
$f_{x}({\mathrm{mm}}^{-1})$	$d_{10}$	$d_{25}$	$d_{50}$	$d_{75}$	$d_{90}$
0.000	0.44	0.89	1.80	3.48	5.91
0.025	0.40	0.79	1.56	2.90	4.77
0.050	0.33	0.65	1.23	2.17	3.42
0.075	0.27	0.53	0.99	1.70	2.61
0.100	0.23	0.45	0.83	1.39	2.10
0.125	0.20	0.39	0.71	1.17	1.76
0.150	0.17	0.34	0.62	1.02	1.51
0.175	0.15	0.30	0.55	0.90	1.33
0.200	0.13	0.27	0.49	0.81	1.19
0.250	0.11	0.22	0.41	0.67	0.98
0.300	0.09	0.19	0.35	0.58	0.84
0.500	0.06	0.13	0.24	0.40	0.58
$\mu_{s}^{\prime }/\mu_{a}=\;50$	Sampling depth (mm)				
$f_{x}({\mathrm{mm}}^{-1})$	$d_{10}$	$d_{25}$	$d_{50}$	$d_{75}$	$d_{90}$
0.000	0.39	0.78	1.54	2.84	4.66
0.025	0.37	0.72	1.40	2.53	4.07
0.050	0.31	0.62	1.16	2.02	3.17
0.075	0.26	0.52	0.96	1.63	2.50
0.100	0.22	0.44	0.81	1.36	2.04
0.125	0.19	0.38	0.70	1.16	1.73
0.150	0.17	0.33	0.61	1.01	1.49
0.175	0.15	0.30	0.54	0.89	1.32
0.200	0.13	0.27	0.49	0.80	1.18
0.250	0.11	0.22	0.41	0.67	0.98
0.300	0.09	0.19	0.35	0.58	0.84
0.500	0.06	0.13	0.24	0.40	0.58
$\mu_{s}^{\prime }/\mu_{a}=\;20$	Sampling depth (mm)				
$f_{x}({\mathrm{mm}}^{-1})$	$d_{10}$	$d_{25}$	$d_{50}$	$d_{75}$	$d_{90}$
0.000	0.33	0.64	1.22	2.13	3.34
0.025	0.31	0.61	1.15	2.01	3.13
0.050	0.28	0.55	1.02	1.75	2.69
0.075	0.24	0.48	0.88	1.49	2.26
0.100	0.21	0.42	0.76	1.28	1.91
0.125	0.18	0.36	0.67	1.11	1.65
0.150	0.16	0.32	0.59	0.98	1.45
0.175	0.14	0.29	0.53	0.87	1.29
0.200	0.13	0.26	0.48	0.79	1.16
0.250	0.10	0.22	0.40	0.66	0.97
0.300	0.09	0.19	0.35	0.58	0.84
0.500	0.06	0.12	0.24	0.40	0.58
$\mu_{s}^{\prime }/\mu_{a}=\;10$	Sampling depth (mm)				
$f_{x}({\mathrm{mm}}^{-1})$	$d_{10}$	$d_{25}$	$d_{50}$	$d_{75}$	$d_{90}$
0.000	0.27	0.54	1.00	1.71	2.61
0.025	0.27	0.52	0.97	1.65	2.51
0.050	0.24	0.48	0.90	1.51	2.28
0.075	0.22	0.44	0.80	1.34	2.01
0.100	0.19	0.39	0.71	1.19	1.77
0.125	0.17	0.34	0.64	1.05	1.56
0.150	0.15	0.31	0.57	0.94	1.39
0.175	0.14	0.28	0.51	0.85	1.25
0.200	0.12	0.25	0.47	0.77	1.13
0.250	0.10	0.21	0.40	0.66	0.96
0.300	0.09	0.18	0.35	0.57	0.83
0.500	0.06	0.12	0.24	0.40	0.58
$\mu_{s}^{\prime }/\mu_{a}=\;\;4$	Sampling depth (mm)				
$f_{x}({\mathrm{mm}}^{-1})$	$d_{10}$	$d_{25}$	$d_{50}$	$d_{75}$	$d_{90}$
0.000	0.21	0.42	0.78	1.31	1.95
0.025	0.20	0.41	0.77	1.28	1.91
0.050	0.19	0.39	0.73	1.22	1.82
0.075	0.18	0.36	0.68	1.13	1.68
0.100	0.16	0.33	0.63	1.04	1.54
0.125	0.15	0.30	0.57	0.95	1.40
0.150	0.13	0.28	0.52	0.87	1.28
0.175	0.12	0.25	0.48	0.80	1.17
0.200	0.11	0.23	0.44	0.74	1.08
0.250	0.09	0.20	0.38	0.64	0.93
0.300	0.08	0.18	0.34	0.56	0.82
0.500	0.06	0.12	0.24	0.40	0.59
$\mu_{s}^{\prime }/\mu_{a}=\;\;1$	Sampling depth (mm)				
$f_{x}({\mathrm{mm}}^{-1})$	$d_{10}$	$d_{25}$	$d_{50}$	$d_{75}$	$d_{90}$
0.000	0.12	0.28	0.56	0.97	1.45
0.025	0.12	0.28	0.55	0.96	1.44
0.050	0.12	0.27	0.54	0.93	1.40
0.075	0.11	0.26	0.52	0.89	1.34
0.100	0.11	0.24	0.49	0.85	1.27
0.125	0.10	0.23	0.46	0.80	1.20
0.150	0.09	0.22	0.44	0.75	1.13
0.175	0.09	0.20	0.41	0.71	1.06
0.200	0.08	0.19	0.39	0.67	1.00
0.250	0.07	0.17	0.35	0.60	0.90
0.300	0.07	0.16	0.32	0.55	0.82
0.500	0.05	0.12	0.24	0.41	0.61
